# Time-restricted eating (TRE) for obesity in general practice: study protocol of a controlled, randomized implementation study (INDUCT) within the Research Practice Network Baden-Wuerttemberg (FoPraNet-BW)

**DOI:** 10.1186/s12937-025-01091-y

**Published:** 2025-03-08

**Authors:** Lorena Braun, Hannah Haumann, Andreas Polanc, Roland Koch, Elke Feil, Anika Klein, Claudia Salm, Frank Peters-Klimm, Gudrun Hübner, Christian Thies, Stephanie Biergans, FoPraNet-B. W. Study Group, Attila Altiner, Annika Baldauf, Anne Barzel, Sibylle Beck, Arndt Becker, Christina Buchta, Sven Dörflinger, Arthur Kraus, Andy Maun, Anna Molle, Karin Scheeser, Patrick Schmutz, Sandra Schramm, Stefanie Joos

**Affiliations:** 1 Institute for General Practice and Interprofessional Care, University Hospital Tübingen, Tübingen, Germany; 2 Institute of General Practice, Medical Center, University of Freiburg, Freiburg, Germany; 3 Department of Primary Care and Health Services Research, Heidelberg University Hospital, Heidelberg University, Heidelberg, Germany; 4 Department of General Practice and Primary Care, Ulm University Hospital, Ulm, Germany; 5 Reutlingen Research Institute, Reutlingen University, Reutlingen, Germany; 6 Medical Data Integration Center (meDIC), University Hospital Tübingen, Tübingen, Germany

**Keywords:** General practice, Obesity, Time-restricted eating, General Practice-Based Research Network, PROMs

## Abstract

**Background:**

Obesity is a major health problem with a significant impact on quality of life and is a major risk factor for other diseases such as diabetes. There is a growing body of evidence that time-restricted eating (TRE) as one form of intermittent fasting (IF) represents a promising weight management strategy. Given the lack of evidence-based weight management strategies in the primary health care setting, the aim of this study is to investigate the effect of TRE in general practice in Germany.

**Methods:**

INDUCT is a type hybrid I, randomized-controlled study conducted in 41 practices within the newly established general practice-based research network (GPBRN) in Baden-Wuerttemberg (FoPraNet-BW). The study population consists of patients with a Body Mass Index between 30–45 kg (kg)/m^2^. The intervention group receives TRE at the scheme 16:8 (16 h fasting; 8 h energy intake) while the control group receives care as usual. The primary outcome is change in body weight under a 6-month period of TRE. Secondary outcomes are related to the patient (e.g. quality of life) and the practice (e.g. knowledge about research in own practice). As the INDUCT study represents one of the first four use-cases within the Research Network Baden-Wuerttemberg (FoPraNet-BW), feasibility is a further secondary outcome. The target sample size is 208 patients with a 1:1 randomization. An intention-to-treat approach is used for data analysis.

**Discussion:**

INDUCT adds evidence on the effect of TRE as a weight management strategy in general practice. Relevant factors for a sustainable and successful implementation in general practice will be revealed and can be applied for future implementation of TRE interventions in general practice if proven successful. In addition, important lessons learned regarding the conduction of clinical research within FoPraNet-BW will be derived. This fosters a sustainable implementation of a research infrastructure in general practice in Germany.

DRKS-ID (German Clinical Trials Register-ID): DRKS00031928 (Date of registration in DRKS: 2023–06-22; Link: https://drks.de/search/de/trial/DRKS00031928).

**Supplementary Information:**

The online version contains supplementary material available at 10.1186/s12937-025-01091-y.

## Introduction

Obesity is a major health problem with a significant impact on wellbeing and quality of life. Also, it is a major risk factor for non-communicable diseases such as diabetes, cardiovascular diseases and certain types of cancer [1–3]. Obesity and obesity-related diseases have a growing impact on health systems as more than 2.6 billion people worldwide were expected to be obese in 2020, according to the World Obesity Atlas. Therefore, the World Health Organization (WHO) states that epidemic proportions regarding obesity are reached constituting a public health crisis [2, 4].

In Germany, the prevalence of obesity has been rising steadily since 1990 [5]. A study showed that in 2019–2020 19% of the German adult population were obese (Body Mass Index (BMI) > 30 kg/m^2^) and 34,5% were overweight (BMI > 25 kg/m^2^—< 30 kg/m^2^) [6]. Nevertheless, there is no specific guideline for the treatment of obesity in Germany. International guidelines for obesity prevention, diagnosis and therapy recommend weight reduction supported by a multidisciplinary team for a BMI > 30 kg/m^2^ and for those with BMI > 25 kg/m^2^ accompanied by additional associated health problems. For individuals with a BMI > 35 kg/m^2^ bariatric surgery is recommended [7].

Part of the “WHO acceleration plan to stop obesity” is the increased integration of the primary health care setting in obesity management while there is a lack of evidence-based approaches [2]. A systematic review showed weight management interventions as part of the service in primary care to be effective in regard to weight loss [8]. Nutritional advice provided by General practices (GPs) showed improved dietary habits and risk factors in patients with lifestyle-related chronic diseases in another systematic review [9]. However, most studies within TRE have recruited participants from other settings such as specialized diabetes clinics and there are few reports on the effectiveness of a TRE approach in GP [8, 10]. Against this background, there is a need for studies on evidence-based weight management strategies specifically tailored to and originating from the primary care sector.

One potential strategy is intermittent fasting (IF), which includes weight management schemes that restrict food intake to certain hours of the day or to certain days of the week. A specific form of IF is time-restricted eating (TRE). TRE does not limit energy intake, but shortens the daily time window for food intake. Fasting periods of 12 to 18 h daily represent common TRE schedules. [11].

Since 2015, there has been a growing body of literature on the effects of TRE in humans. A Cochrane review from 2021 shows that different forms of short-term IF (three months) reduce body weight compared to usual eating habits [12]. However, the results of the individual studies are heterogenous, examining different populations, outcomes, TRE schemes and durations of the intervention. A review by Petersen et al. shows high adherence (80%—90% of days recorded) to TRE interventions with a significantly greater weight loss under TRE for 8–12 weeks. The review also states that with the present data, the knowledge about cardiometabolic effects of TRE in overweight or obese individuals is limited [11]. Another systematic review by Adafer et al. supports this finding showing positive effects regarding different metabolic outcomes [10]. Both reviews call for long-term studies to evaluate TRE, especially for studies on Patient Reported Outcome Measures (PROMs) [10, 11].

So far there are few studies on feasibility and effectiveness of TRE outside the hospital setting. A study from Switzerland involving 54 community-based adults with at least one component of metabolic syndrome showed that a 12-h daily fasting period led to a 1.6% reduction in body weight over six months compared to usual diet [13]. To our knowledge, there is only one uncontrolled pilot study with a pre-post design from GP in Germany. The 38 participants in that study fasted for 15 to 16 h on 85,5% of the days recorded, with an inverse relationship between fasting duration and the amount of weight loss. After following the TRE scheme for three months participants experienced moderate weight loss (− 1.7 ± 2.5 kg) and corresponding changes in BMI (− 0.6 ± 0.9 kg/m^2^), but lost − 5.3 ± 3.1 cm waist circumference. Weight loss and changes in BMI correlated with decreases in glycated hemoglobin (HbA1c), the latter being diminished by -1.4 ± 3.5 mmol/mol (*p* = 0.003) [14]. In a secondary analysis, this study was compared to a study on TRE in healthy employees at the University of Ulm. In this comparison, participants in the GP practices showed a greater reduction in waist circumference and waist-to-height ratio [15].

The primary aim of the INDUCT study (Intermittent fasting for obesity in general practice—a controlled, randomized implementation study within the General Practice-Based Research Network (GPBRN) Baden-Wuerttemberg) is to examine the effect of a 16:8 TRE scheme for six months on body weight in an obese study population from GP. Secondary aims include evaluating the effects of the 16:8 TRE scheme on clinical and laboratory outcomes (e.g. blood pressure, metabolic parameters) as well as PROMs (e.g. quality of life, sleep quality). The feasibility of the trial within the newly established FoPraNet-BW including the usability of the developed digital research infrastructure represents a further (secondary) outcome.

## Methods

### Study design and setting

The type I hybrid, randomized-controlled study is conducted within FoPraNet-BW [16, 17]. FoPraNet-BW is a GPBRN that has been established across the federal state of Baden-Wuerttemberg, Germany since 2020 [funding code 01GK1904A-D]. FoPraNet-BW is an association of general practitioners and their practice teams, four university departments of General Practice from Baden-Wuerttemberg (Tuebingen, Freiburg, Heidelberg and Ulm) and the Reutlingen research institute. A digital research infrastructure was designed and practice teams underwent a research training program enabling them to conduct research in their own practices [18]. This research training program was designed by FoPraNet-BW according to the research ready concept of the DESAM ForNet (Initiative of German Practice-Based Research Networks) [19]. 41 GP practices, called “research practices”, participated in the INDUCT study. The total duration of the study was 18 months (July 2023 – November 2024).

**Fig. 1:**
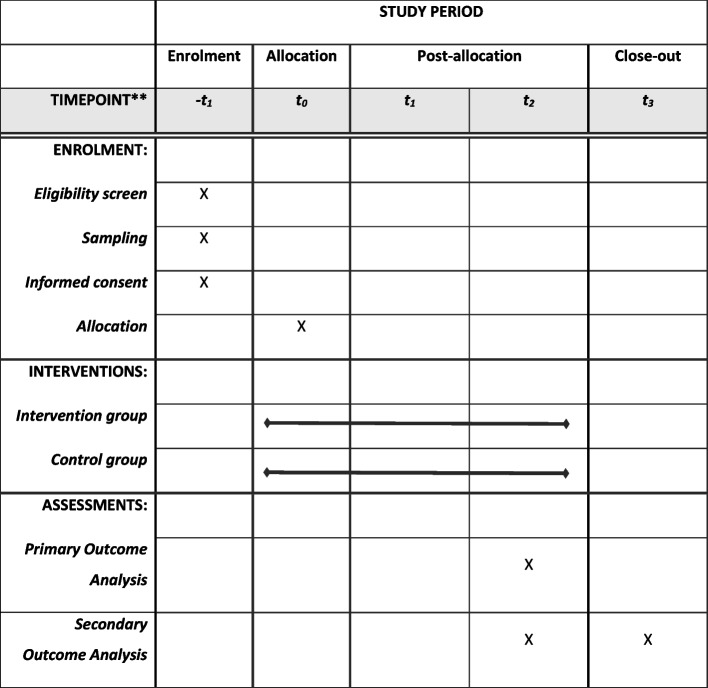
Schedule of enrolment, interventions and assessments ** Between t0 and t1 the intervention group has two additional visits in the GP practice as well as one phone-visit to support the beginning of TRE

The type I hybrid research approach of this study addresses the interface between clinical research and implementation research: The clinical effects of the 16:8 TRE regimen over a period of 6 months are confirmatory tested while observing and gathering information on its implementation. This study design is chosen as INDUCT represents the first use case of FoPraNet-BW. A comprehensive mixed-methods process evaluation based on the Consolidated Framework for Implementation Research (CFIR) covers aspects about the implementation process from all participants involved (e.g. patients, practice personnel, scientific staff) [20]. The results of the process evaluation will be reported in a separate publication. The SPIRIT (Standard Protocol Items: Recommendations for Interventional Trials) 2013 statement was used in this study protocol [21]. The protocol includes the SPIRIT figure template (Fig. 1). The SPIRIT 2013 checklist of recommended items to be addressed is provided in Supplementary File A.

### Outcomes

The aim of the INDUCT study is twofold: first the effectiveness of TRE for obesity in a GP population is examined. Therefore, the primary outcome is change in body weight under a 6-month period of TRE. Secondary outcomes can be divided into outcomes related to the participants and outcomes related to the practices (see Table 1). Furthermore, the feasibility of conducting a controlled, randomized clinical trial within the newly established FoPraNet-BW is examined. Criteria to assess feasibility include the number of practices participating in the study, the participation rate at patient and practice level.

**Table 1:** Primary and secondary outcomes

Primary outcomes	Secondary outcomes	
	**Participant related goal criteria**	**Goal criteria on practice level**
**TRE**: • Change in body weight	**Clinical goal criteria:** Change in … • … metabolic parameters (blood lipids: high-density lipoprotein, low-density lipoprotein, total cholesterol, triglycerids; fasting blood glucose, HbA1c, fasting blood insulin levels) • … insulin resistance (HOMA-IR) • … blood pressure • … waist/abdominal circumference • … waist-to-hip-ratio • … BMI **PROMs** • World Health Organization- Five Well-Being Index [22] • Quality of life (European Quality of Life 5 Dimensions 5 Level Version) [23] • Sleep quality (Pittsburgh Sleep Quality Index) [24] • Patient activation (Patient Activation Measure 13) [25] • Psycho-social distress due to external appearance, body image, expectations of intervention, physical function/discomfort, social participation, body satisfaction (Body-Q) [26] • Physical activity (International Physically Activity Questionnaire) [27] • Adherence to TRE • Timing of food intake • Feeling of hunger • Quality of nutrition (Nutritional pattern index) [28]	Increased knowledge regarding • Obesity^a^ • Knowledge of interventions for obesity in GP^a^ • Knowledge of research in own GP practice^a^ • Knowledge of how to implement intervention studies in own GP practice^a^ • Own attitudes towards obesity^a^

^a^Outcome is assessed by a mixed method process evaluation

### Intervention

The intervention group (IG) follows a 16:8 TRE scheme (8-h meal period followed by a 16-h fasting period) for 6 months. Participants were instructed and supported by members of the participating practice teams, usually a GP practitioner and a medical assistant, called “research tandem”. The research tandem was provided with two 90 min webinars on TRE, which were developed in collaboration with a team of TRE experts. Both webinars were recorded and made accessible to all research tandems during the entire study period. Participants were provided with an information sheet with the most important facts about TRE. There was no recommended diet for the eating period. During the fasting period participants could drink energy-free beverages such as tea or coffee without milk or sugar. The start time of the eating period could be chosen individually by the study participants on a daily basis. The control group (CG) received usual care for obesity in their GP practice.

To address adherence in both groups, study diaries were used to document adherence to the intervention and challenges during the intervention phase (5 diaries of 1 week each, one before the beginning of the study and the other ones in week 5, 11, 17 and 23). Furthermore, at the start of TRE the IG also had two additional visits in the GP practice and one phone-visit, to support the early phase of the intervention.

Prior studies on TRE did not show safety-relevant events. Possible adverse effects of TRE are: nausea, vomiting, feeling unwell, diarrhea, constipation, headache, migraines, sleep disorders or changes in sleep rhythm [11]. In the present investigation adverse events are documented by the practice teams via the digital research infrastructure. The principal investigator is informed by means of the digital infrastructure that an adverse event is recorded and needs to consider possible consequences.

### Sample size

The target sample size for the INDUCT study is 208 patients with obesity based on previous studies, which stated that the clinically significant difference in body weight loss up to 24 weeks is 2.5%. The change in body weight of 2.5% corresponds approximately to ~ 2.2 kg for INDUCT participants (BMI ≥ 30 kg/m^2^) with an estimated average height of 172 cm [29]. Based on two studies, a standard deviation of 5 kg is assumed for within-group changes [8, 30]. A two-tailed independent t-test (alpha = 0.05; effect size d = 0.44) with statistical power of 0.8 was used to calculate the sample size of n = 83 per group (n = 166 total) to detect a difference in weight change of 2.2 kg (standard deviation: 5 kg) between the two groups. With a 20% dropout-rate, a total of n = 208 participants need to be recruited. Therefore, the aim was to recruit 4 to 5 participants from each participating research practice for the INDUCT study.

### Recruitment process, inclusion/exclusion criteria and study flow

**Fig. 2:**
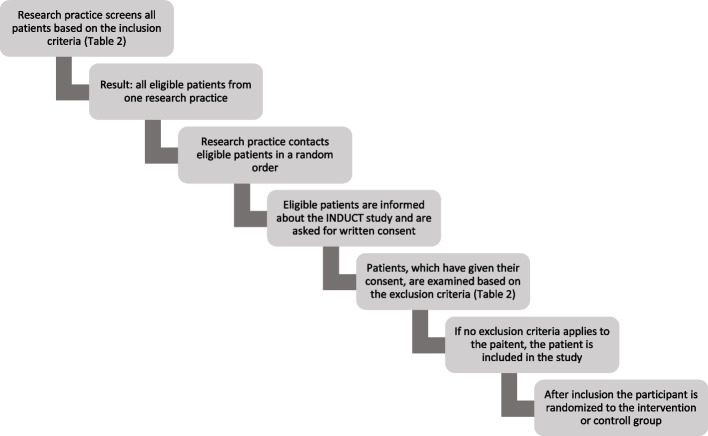
Recruitment process

Study participants are recruited from the participating research practices in FoPraNet-BW. All patients receiving care from one of the participating practices are potentially eligible for participation. The recruitment process is shown in Fig. 2. The main inclusion criterion was a BMI between 30–45 kg/m^2^. All inclusion and exclusion criteria are listed detailed in Table 2.

**Table 2:** Inclusion and exclusion criteria

Inclusion criteria
o BMI ≥ 30—≤ 45 kg/m^2^ or ICD-10: E66
o ≥ 18 years and ≤ 65 years
o Regular care provided in recruiting research practice
o Capacity to consent
o No objection to data processing for the purpose of identifying potential study participants (case finding)
**Exclusion criteria (after study-specific informed consent and consent to data processing is signed)**
o Pregnancy (up to 6 months post-partum) or lactation period
o History for or presence of an eating disorder: anorexia, bulimia, binge eating disorder
o Presence of uncontrolled hyper-/hypothyroidism
o Current presence of an organic or symptomatic mental disorder as assessed by the study physician
o Diabetes mellitus type 1 or diabetes mellitus type 2 requiring insulin therapy
Anamnestic evidence for: o Cardiovascular event within the past 12 months (acute coronary syndrome, cardiac catheterization, bypass surgery, hospitalization due to heart failure, stroke, transient ischemic attack) or uncontrolled arrhythmia o Active or therapeutically unstable (insufficiently achieved symptom control) or expected risk of worsening of the underlying disease under TRE in diseases from the fields of rheumatology, oncology, hematology, infectiology, gastroenterology, psychiatry, nephrology or endocrinology o Uncontrolled arterial hypertension o Current harmful use of illegal substances and/or alcohol o Performed bariatric surgery o Current or within the past 6 months use of medications which, according to the assessment of the study physician, favor obesity or influence body weight and affect energy and glucose balance (e.g. steroids, antiepileptic drugs, psychotropic drugs, appetite suppressants) as well as immunosuppressants
Pathological results of laboratory examination at screening: o Advanced renal insufficiency (glomerular filtration rate (GFR) < 45 ml/min or assessed or known to be so by study physician) o Advanced hepatic insufficiency (elevation of transaminases above 3 times normal or assessed or known to be so by study physician) o Anemia o Additional laboratory parameters assessed by the study physician as critical for study participation
o History of or existing retinal detachment
o Presence of any secondary form of obesity (incl. polycystic ovary syndrome, Cushing's syndrome, Prader-Willi syndrome, hypogonadism, Lauren-Moon-Biedl-Bardet syndrome, acromegaly, hypothalamic-pituitary hormone axis disorders)
o Physician-ordered other form of diet (e.g. for celiac disease)
o Participation in a weight loss program with weight loss of more than 5% body weight in the past 3 months
o Lack of suitability for study participation assessed by the study physician
o Concurrent participation in another intervention study or participation in another intervention study within the last 3 months

### Randomization

A 1:1 randomization at patient level is performed by a programmed, automatic randomization tool provided by the research institute in Reutlingen as a part of the digital research infrastructure. Through this tool the research tandem assigns the patients to the groups. No blinding is applied.

### Data collection methods

**Fig. 3:**
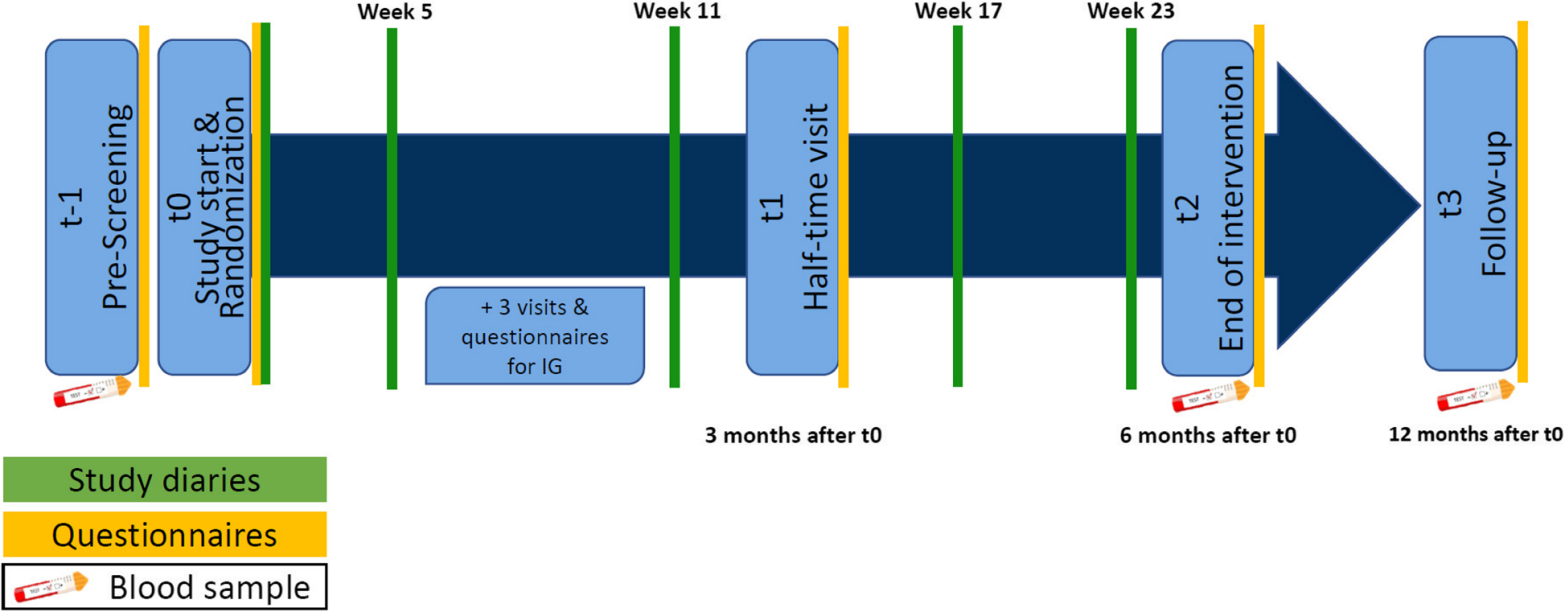
Overview of the course of the study

Within FoPraNet-BW, a federated data processing approach was implemented [18]. The research tandem and the participants, themselves, enter data. In INDUCT, data is entered directly into an electronic data capture system (Research Electronic Data Capture (REDCap)) via the digital research infrastructure. Research tandems can enter the electronic data capture system browser-based from their practices. Participants enter the data token-based, with tokens handed through the research tandems (see Fig. 3 and Tab. 3). All patient identifying information remain within the research practice. On consent to study participation and start of data entry, a pseudonymization process is started with the identification list remaining within the practice. The participating university departments can monitor data entry as they can access the data capture system being provided with the pseudonymized information only. Furthermore, the university departments can not change the pseudonymized data. All needed material for data collection (questionnaires, checklists etc.) are provided to the participating practices through the digital research infrastructure by the university departments.

**Table 3:** Content and time of data collections

Point of time	**Research tandem**	**Study participant**
t-1 Week -1 Pre-screening	Data collection form (Study information and consent, participants age, participants sex, inclusion/exclusion criteria, blood sample, laboratory examination, study inclusion, randomization, documentation physical examination)	
t0 Week 0 Study start	Data collection form practice (participants current health situation^a^)	• Study diary^b^ • Self-developed Questionnaire (including sociodemographics, current health situation and previous weight management experiences) • Standardized patient questionnaires***
Week 1	Data collection form Visit (IG only) (blood pressure, heart rate, parameters, other examination finds, (serious) adverse events, other aspects of the visit)	
Week 2	Data collection form Visit (IG only) (blood pressure, heart rate, other examination finds, adverse events, other aspects of the visit)	
Week 5		Study diary^b^
Week 6	Data collection form Phone-Visit (IG only) (adverse events, other aspects of the visit)	
Week 11		Study diary^b^
t1 Week 12 Half-time visit	Data collection form practice (participants current health situation^a^, laboratory examination)	• Self-developed Questionnaire (including previous weight management experiences) • Standardized patient questionnaires^c^
Week 17		Study diary^b^
Week 23		Study diary^b^
t2 Week 24 End of intervention	Data collection form practice (participants current health situation^a^, laboratory examination)	• Self-developed Questionnaire (including sociodemographics and previous weight management experiences) • Standardized patient questionnaires^c^
t3 Week 48 Follow-up	Data collection form practice (participants current health situation^a^)	• Self-developed Questionnaire (including sociodemographics and previous weight management experiences) • Standardized patient questionnaires^c^

^*a *^The questionnaire about the participants current health situation contains current diseases, performed operations, current medications, current body mass index calculated from body weight and height, hip and abdominal circumference. At t1, t2 and t3 laboratory parameters are assessed (cholesterol, high-density lipoprotein, low-density lipoprotein, triglycerides, blood sugar, HbA1c, C-reactive protein, insulin)

^b^ Study diaries contain questions over 7 days about the current health situation, attitude towards time-restricted easting (IG only), the study, eating habits (CG only), time of the first and last meal a day, time-restricted eating (IG only), physical activity and a field for other feedback

^c^ The standardized patient questionnaires contain the German versions of the following standardized questionnaires: European Quality of Life 5 Dimensions 5 Level Version [23], World Health Organization-Five Well-Being Index [22], Pittsburgh Sleep Quality Index [24], Patient Activation Measure 13 [25], International Physically Activity Questionnaire [27], Body-Q [26], Nutritional pattern index [28])

All participants fill out study diaries and answer questionnaires at defined points of time during the study period. As mentioned, all data can be entered in an electronic data capture system (REDCap database) directly by the participants via a token-based access to the database. In case of lack of access to the REDCap database paper-based data forms were used. As INDUCT does not represent a clinical study under the regulation of drug studies and is a non-blinded study with little safety risks, no data monitoring committee was established.

### Data analysis (Data management and statistical analyses)

The four university departments of general practice and the Reutlingen research institute as well as the medical data integration center (meDIC) at the University Hospital Tuebingen can access the final data set in REDCap for analysis. The meDIC will store the data sets on a dedicated project virtual machine (VM) in the clinical network of the university hospital Tuebingen. The VM can be accessed by dedicated analysts using an integrated user management. It is equipped with R and python for analysis [31, 32]. After finalization of the study and analysis the VM with all data sets, data quality reports, analysis results and scripts will be archived for a period of 10 years for reproducibility.

A data quality report will be generated by the meDIC at several timepoints during the study period to identify data quality issues (e.g. missing data, data out of range, wrong data formats). The data quality report will be used to document and correct data quality issues before analysis.

Besides descriptive statistics an intention-to-treat approach is used for data analysis. The primary end point for analysis is the change in body weight or BMI. A machine learning approach will be used to investigate predictors for weight change in patients. The focus will be on explainable machine learning (ML) by using glassbox models, such as explainable boosting machines, and explainability techniques for blackbox models [33, 34]. Explainable machine learning allows a deeper understanding of the influence of individual variables in the model. Variables with more than 30% missing values will be excluded from ML-analysis.

Several secondary end points, such as changes of metabolic factors or blood pressure, will primarily be investigated using descriptive statistics. Statistical tests for significance will be used and corrected for multiple testing, if necessary. Individual secondary end points might also be analyzed using a ML-approach. Feasibility outcomes will be assessed and analyzed via descriptive process data from the digital research infrastructure.

## Discussion

### Strengths and challenges

Systematic reviews assessing previous studies on TRE show positive effects on weight loss but there is limited knowledge about the effects on metabolic outcomes and PROMs as the latter are scarcely addressed [10, 11, 33, 35]. Furthermore, evidence from the primary care sector is lacking, and most studies have a duration of 8 to 12 weeks only, while the evaluation of adherence in weight loss approaches is crucial. Therefore, studies on the long-term effects of TRE are needed [10]. Patient populations in the primary care sector represent an unselected patient population as well as a broad spectrum of patients. Recruitment in primary care is advantageous, as this sector is highly relevant in terms of disease burden and social relevance in obesity. The INDUCT study addresses these research gaps by including PROMs such as sleep quality, feeling of hunger and quality of life. Also, the duration of the intervention in the INDUCT study is six months, with a 6-month follow-up giving the possibility to derive long term-effects of TRE.

Challenges in this study arose from the heterogeneity of the participating research practices and in consequence of the research tandem. INDUCT is the first study and first randomized controlled trial within the newly established research infrastructure FoPraNet-BW. While all participating practice teams have undergone a training on research in their practice, there is none to little experience in conducting a study in their own practice. Also, previous experiences with TRE differ a lot among participating practice teams. We propose that there is a learning curve within the research tandem regarding e.g. support and monitoring of study participants as well as data collection and management. All the named aspects could influence data quality. As every participating practice represents one study center within INDUCT, monitoring of the study prerequisites other approaches than monitoring a RCT with one study center only. Study nurses at the four participating departments of general practice therefore established new processes and made use of the digital research infrastructure.

Furthermore, we hypothesize that the handling of the newly developed software could differ between practices with effects on data quality and recruitment of patients for the study. The development of the digital research infrastructure is considered a constant process as software development continues during the entire duration of FoPraNet-BW based on feedback from all user groups.

As there is no structured treatment approach for obesity in GP in Germany, treatment as usual is difficult to define and to standardize. Therefore, treatment in the CG is expected to show a high variation, which will have an influence on the study results. In knowledge of this, we aimed to address aspects of care in the CG through the study diaries. Another major challenge is to retain participants assigned to the CG. We hypothesize that motivation to start the study is also a motivation for weight reduction, so there is a potential risk that participants from the CG try other weight management approaches. CG participants will all be offered to try TRE after the intervention phase of the INDUCT study and will have the opportunity to be supported by their GP practice. Loss to follow-up is expected in both groups. This loss of study participants is addressed by adding a 20% dropout-ratio into the sample size calculation.

### Patient and public involvement

The study diaries and questionnaires for participants and research practices were developed and piloted in a participatory process with the target groups. Information about the INDUCT study was provided to the research practices and the study participants in FoPraNet-BW via the project website [36], newsletters and the websites of the participating university institutions of general practice. Further information was provided to GP practice teams at CME-events such as the “Day of General Practice”.

The results of the INDUCT study will be presented and discussed with the participating practice teams and the expert advisory board, consisting of representatives from the healthcare sector and patient organizations. Furthermore, the results will be presented and discussed with the Citizens' Advisory Council as well as patient representatives. The Citizens’ Advisory Council was established in 2023 at the Institute of general practice at Freiburg university aiming to integrate the perspectives and needs of citizens concerning research in GP. Results from these discussions will be included in the dissemination of the study results. In order to inform both the general public and the medical community about the results of the INDUCT study, publications are planned via open access publications, the FoPraNet-BW project website and the website of the Initiative of German GPBRNs DESAM ForNet [36, 37]. Publications in simple lay language are also planned in collaboration with the Citizens' Advisory Board and patient representatives.

### Ethical considerations and data protection aspects

The INDUCT study was approved on 01.03.2023 by the Ethics Committee of the Medical Faculty of Tuebingen (097/2023BO2). The digital research infrastructure as part of the PBRN FoPraNet-BW was approved prior to the start of INDUCT in a consensual process among all ethics committees of the participating medical faculties at the participating universities under the leadership of the Baden-Wuerttemberg Medical Association (F-2022–059/ coord. B).

A comprehensive data protection concept for FoPraNet-BW was developed together with and approved by the data protection officers of the participating university hospitals (Tuebingen, Freiburg, Heidelberg, Ulm) and the University of Reutlingen. Part of the federated data infrastructure is that study teams at the university departments have access to pseudonymized data only, all personal data remains within the participating practices. In order to be able to collect data as part of the study, the study participants had to give their informed consent in their GP practice to participate.

### Trial status

At the beginning of the INDUCT study 41 research practices were part of the FoPraNet-BW. The recruitment process of the participants in these research practices was started in August 2023 and completed in December 2023. 149 eligible participants have been recruited in those 41 research practices. 12 months after the study inclusion a follow-up is planned. The last patient-out therefore is expected in December 2024.

### Outlook

If TRE with the 16:8 scheme proved to be successful in regard to weight loss, metabolic aspects and further PROMs, while being safe, it can represent a concept that could be transferred to care in GP in Germany. Furthermore, the accompanying process evaluation is expected to identify facilitating factors and barriers that may be crucial for further implementation of TRE. In addition, valuable insights from conducting research within a GPBRN in Germany can be derived. One focus of the evaluation is also the development of strategies to improve recruitment of patients in their practices. Lessons learned from INDUCT can be used to plan further intervention studies with the research practices of the FoPraNet-BW and intervention studies in other GPBRN in general. Also, further intervention studies on weight management in GP practices could be planed based on the results of this study.

## Supplementary Information

Supplementary Material 1.

## Data Availability

No datasets were generated or analysed during the current study.

## Abbreviations

BMBF: Federal Ministry of Education and Research (Germany)
BMI: Body-Mass-Index
CFIR: Consolidated Framework for Implementation Research
CG: Control Group
CME: Continuing Medical Education
DESAM ForNet: Initiative of German Practice-Based Research Networks
DRKS: German Clinical Trials Register
FoPraNet-BW: Research Network Baden-Wuerttemberg/ Forschungspraxennetz Baden-Württemberg
GFR: Glomerular filtration rate
GP: General practice
GPBRN: General practice-based research network
HbA1c: Glycated hemoglobin
IF: Intermittent Fasting
IG: Intervention Group
INDUCT: Intermittent Fasting with obesity in general practice
kg: Kilogram
m^2^: Square meters
meDIC: Medical Data Integration Center
PROMs: Patient Reported Outcome Measures
REDCap: Research Electronic Data Capture
SPIRIT: Standard Protocol Items: Recommendations for Interventional Trials
TRE: Time-restricted eating
WHO: World Health Organization
